# PLIN1 associates with lipid droplets and mediates lipolysis in the perirenal brown adipose tissue of yak calves

**DOI:** 10.3389/fvets.2026.1710538

**Published:** 2026-04-13

**Authors:** Zhenshuo Bai, Weifeng Fan, Lizhuang Hao, Ali Mujtaba Shah, Fuju Chen, Jiaoyang Du, Jinqi Luo, Yiying Yin, Yu Hu, Yiran Tong

**Affiliations:** 1 College of Agriculture and Animal Husbandry, Qinghai University, Xining, China; 2 Key Laboratory of Plateau Grazing Animal Nutrition and Feed Science of Qinghai Province, Qinghai University, Xining, China; 3 Guangdong Provincial Key Laboratory of Animal Nutrition and Regulation, College of Animal Science, South China Agricultural University, Guangzhou, China

**Keywords:** brown adipose tissue, lipid droplet, lipolysis, Perilipin 1, perirenal adipocytes, white adipose tissue, yak

## Abstract

We previously reported that perirenal adipocytes in yak calves undergo a remarkable transformation during the early postnatal period (1–30 days), during which lipid droplets (LDs) consolidate from numerous small multilocular structures into a single unilocular droplet. However, the molecular mechanisms underlying this developmental transition remain poorly understood. Perilipin 1 (PLIN1), which is highly expressed in adipocytes, promotes the formation of unilocular LDs and plays a critical role in regulating lipolysis. Based on these findings, we aimed to elucidate the role of PLIN1 in unilocular LD formation in perirenal adipocytes. PLIN1 expression and localization were analyzed in perirenal adipose tissue of yak calves at different postnatal stages (1 and 30 days) and during *in vitro* adipocyte differentiation. Functional roles of *PLIN*1 were assessed using inhibition experiments to evaluate its effects on lipid droplet morphology, lipid accumulation, and the expression of lipolysis-related genes and proteins. In addition, cold exposure experiments were performed to examine changes in thermogenic and lipolytic markers. The results of the current study revealed that *PLIN*1 was localized in both brown and white adipocytes, with significantly higher mRNA and protein expression levels in 30-day-old calves than in 1-day-old calves. During in vitro perirenal adipocyte differentiation, *PLIN*1 mRNA expression peaked on day nine. Notably, inhibition of *PLIN*1 led to reduced LD size and lipid accumulation, elevated free fatty acid (FFA) content, and upregulation of mRNA and protein expression of adipose triglyceride lipase (ATGL) and hormone-sensitive lipase (HSL). Furthermore, cold exposure increased the expression levels of *PLIN*1, *HSL*, *ATGL*, *UCP*1, *PPARα*, *PGC*-1*α*, and *COX*7*A*1, and elevated UCP1 protein abundance. In conclusion, our findings indicate that PLIN1 mediates LDs growth and lipolysis in the perirenal adipocytes of yak calves, thereby providing theoretical insights into the molecular mechanisms governing the brown-to-white phenotypic transition during early postnatal development in yaks.

## 1 Introduction

The yak (*Bos grunniens*), a unique and rare species endemic to the Qinghai-Tibet Plateau region, has adapted remarkably well to extremely cold and hypoxic conditions (1). Female yaks typically give birth in March, with the peak calving season occurring between May and June. During this period, pastures remain covered with snow, and the average ambient temperature frequently falls below 0 °C (2, 3). Newborn yak calves walk within approximately 10 min of birth and quickly integrate into the herd. This ability reflects a well-developed capacity for adaptive thermogenesis, which allows them to cope with extra-uterine cold environment (4). However, the specific mechanisms underlying the adaptive thermogenesis in yak calves are poorly understood. Elucidating these cold adaptation strategies is essential not only for preserving the stability of the plateau ecosystem but also for providing an ideal model to investigate biological adaptations to cold environments.

Adipose tissues are the largest reservoirs of energy in the yak body and play a critical role in thermoregulation and energy homeostasis. In mammals, it is primarily classified into two main types: brown adipose tissue (BAT) and white adipose tissue (WAT). Both contain lipid droplets (LDs), but they differ significantly in structure and function. Mature white adipocytes are characterized by a large size, unilocular LD, and function mainly to store excessive energy such as triglycerides (TG) and release free fatty acid (FFA) during energy deficiency (5). In contrast, brown adipocytes contain numerous smaller, multilocular LDs that serve as primary sites of non-shivering thermogenesis (NST). This process is mediated by mitochondrial uncoupling protein 1 (UCP1), peroxisome proliferator-activated receptor alpha (PPARα), and peroxisome proliferator-activated receptor-γ coactivator-1α (PGC-1α) (6, 7), and it enables BAT to generate heat and play a vital role in neonatal cold adaptation (5). Studies have also identified a third type of inducible thermogenic adipocyte, termed beige adipocyte, which emerges within WAT in response to cold or β-adrenergic stimulation. Beige adipocytes exhibit a thermogenic capacity, but the degree to which their properties and functions overlap with those of classical brown adipocytes remains debated (8).

Our previous study demonstrated that brown adipocytes in yak calves transform into white adipocytes within 30 days of birth (9). This morphological shift was consistent with observations in other ruminants, including calves (10–12) and goats (13, 14). However, the mechanisms underlying LD growth and formation of unilocular LD in postpartum yak calves remain poorly understood. The adipocyte-specific Perilipin, ADRP (adipose differentiation-related protein), and TIP47 family (PAT) of LD-associated proteins plays structural and/or regulatory roles in the formation of large LDs.

Perilipin1 (PLIN1), the most extensively studied member of the PAT family, plays a dual role in regulating lipolysis (15, 16). It suppresses basal lipolysis under resting conditions but stimulates lipolysis such as during β-adrenergic stimulation or cold exposure (17–19). Under normal conditions, knockdown or ablation of PLIN1 leads to smaller LDs and reduced triglyceride accumulation, as observed in bovine (20) and chicken adipocytes (21), as well as in WAT in mice, where it confers resistance to diet-induced obesity (22). Conversely, PLIN1 overexpression results in larger LDs with high triglyceride content, as demonstrated in bovine (20) and chicken models (21). Moreover, PLIN1 knockdown/ablation increases the number of key lipases such as adipose triglyceride lipase (ATGL) and hormone-sensitive lipase (HSL). This suggests that PLIN1 restricts basal lipolysis by limiting access of these lipases to the LD surface and, in turn, stabilizing lipid storage and preventing excessive energy consumption.

Upon β-adrenergic stimulation or cold exposure, catecholamines bind to β-adrenergic receptors and, in turn, activate adenylate cyclase and increase cyclic AMP (cAMP) concentrations. This activates protein kinase A (PKA), which phosphorylates PLIN1. Phosphorylated PLIN1 undergoes a conformational change that relieves its inhibition of ATGL and HSL and triggers robust lipolysis (23, 24). Deficiencies in ATGL or HSL impair cold-induced thermogenesis in brown adipocytes during fasting, underscoring their essential role in BAT-mediated thermogenesis.

**Figure 1:**
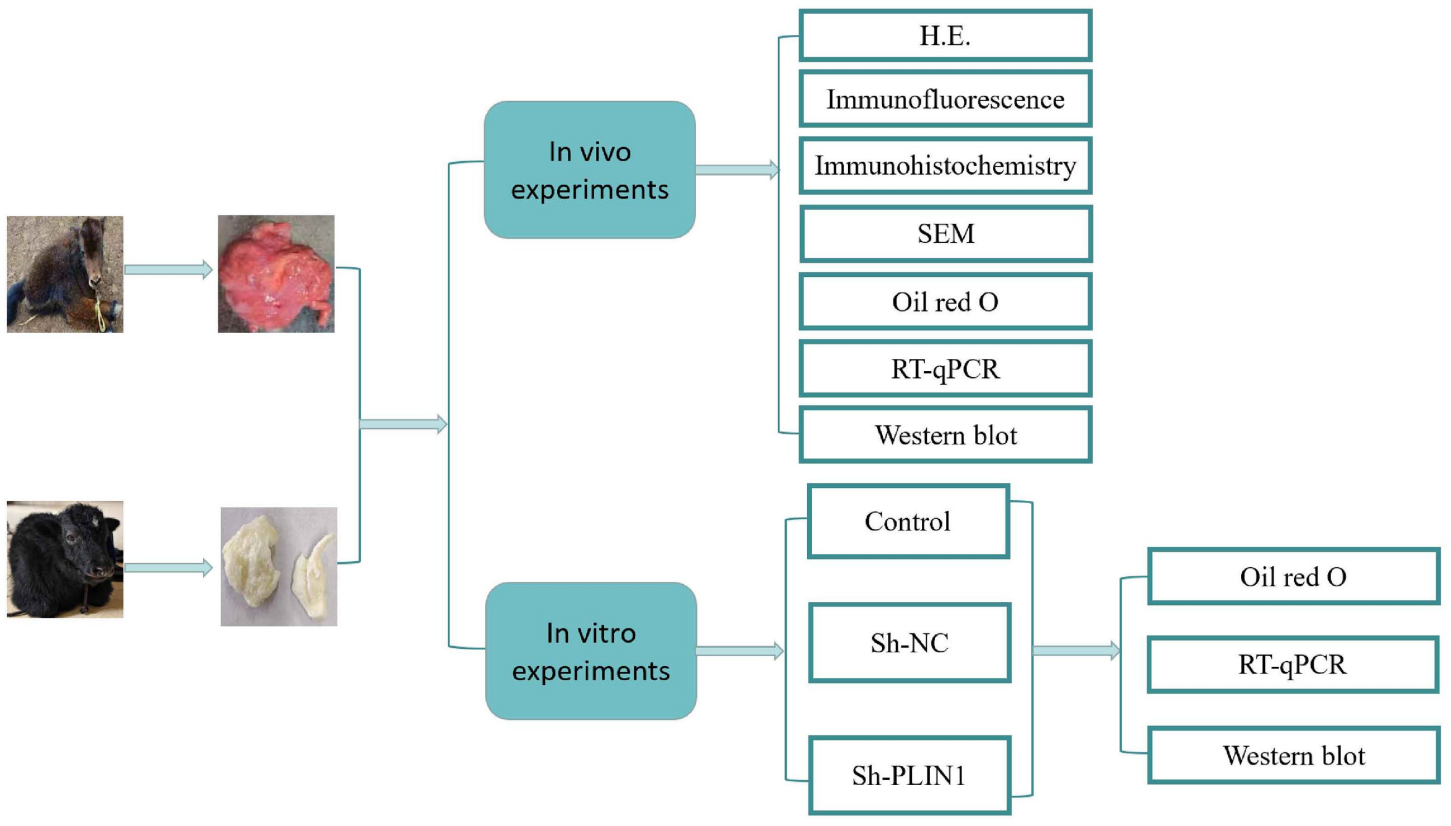
Schematic diagram of the experimental workflow. The study consisted of *in vivo* experiments using perirenal adipose tissue from yak calves and *in vitro* experiments using primary yak preadipocytes. *In vivo* analyses included hematoxylin and eosin (H&E) staining, immunofluorescence, immunohistochemistry, scanning electron microscopy (SEM), Oil Red O staining, Reverse Transcription Quantitative Polymerase Chain Reaction (RT-qPCR), and Western blot. *In vitro* experiments included control, sh-NC, and sh-PLIN1 groups, followed by Oil Red O staining, RT-qPCR, and Western blot analyses.

Thus, PLIN1 is a key regulator of LD morphology and lipid metabolism. However, the molecular mechanisms through which PLIN1 mediates these processes in yak calf adipocytes remain unknown and have not been previously reported. In this study, we examined the distribution and expression of endogenous PLIN1 in the perirenal adipose tissue of 1- and 30-day-old yak calves and correlated them with LD size. We also investigated the effects of PLIN1 on LD size, fat accumulation, FFA content, and the expressions of HSL and ATGL. Further, we evaluated the expressions of PLIN1, HSL, and ATGL in response to the cold treatment. Our findings provide new insights into the mechanisms underlying the conversion of brown to white adipocytes in postnatal yak calves. The experimental design is illustrated in Figure 1.

## 2 Materials and methods

### 2.1 Experimental animals and sample collection

Yak calves were obtained from the Datong Yak Breeding Centre (3,100 m above sea level; 37.25° N, 101.40° E) in the Qinghai Province, China. In April 2024, eight healthy male yak calves were selected and divided into two age groups: 1-day-old (*n* = 4) and 30-day-old (*n* = 4), with average body weights of 15.21 kg and 30.12 kg, respectively. The mean ambient temperature during sample collection was approximately 1 °C.

Yak calves were euthanized by jugular vein injection with a lethal dose of sodium pentobarbital (80 mg/kg body weight; Beuthanasia-D Special; Intervet Inc., Union, NJ, USA). Perirenal adipose tissue was immediately collected after euthanasia. Portions of the tissues were fixed in 4% paraformaldehyde in 0.1 M phosphate-buffered saline (PBS, pH 7.4) or 2.5% glutaraldehyde for histological and ultrastructural observations. The remaining tissues were snap-frozen in liquid nitrogen for reverse transcription quantitative PCR (RT-qPCR), western blot analysis, isolation, and primary culture of preadipocytes. For histological and molecular analyses, samples from each animal were analyzed independently, and each animal was considered an independent biological replicate.

### 2.2 Isolation, culture, induced differentiation, and Oil Red O staining of yak perirenal preadipocytes

Primary yak preadipocytes were isolated from four healthy 3-day-old yaks, according to established protocols (25, 26). Briefly, perirenal adipose tissues were aseptically collected from healthy calves and rinsed with 75% ethanol before being minced into approximately 1 mm^3^ fragments. The tissue fragments were digested with collagenase type I at 37 °C for 60–90 min under constant agitation. The resulting digesta was filtered through a 40 μm nylon mesh and centrifuged at 1400 × *g* for 5 min. The cell pellet was resuspended in an appropriate volume of complete Dulbecco’s Modified Eagle’s Medium (DMEM/F12; Gibco, Shanghai, China) and cultured at 37 °C under 5% CO_2_.

After contact inhibition for 2 days, the culture medium was replaced with an induction cocktail consisting of DMEM/F12 supplemented with 10% FBS (PAN-Biotech, USA), 1% penicillin/streptomycin, 0.5 mM IBMX (Sigma, St. Louis, MO, USA), 1 μM dexamethasone (DEX; Sigma, St. Louis, MO, USA), and 5 μg/mL bovine insulin (Sigma, St. Louis, MO, USA) to initiate differentiation for approximately 9 days. Following differentiation, the cells were stained with saturated Oil Red O solution for 30 min at room temperature, washed thrice with distilled water, and imaged using a light microscope. The mRNA expression levels of PLIN1 were analyzed on days 3, 6, and 9 of differentiation.

### 2.3 Interference of PLIN1 in yak perirenal preadipocytes

The yak PLIN1 gene sequence (GenBank No. XM_021231650.2), a specific short hairpin RNA targeting PLIN1 (sh-PLIN1), and a negative control shRNA (sh-NC) were designed and synthesized by Beijing Tsingke Biotechnology Co., Ltd. Yak perirenal preadipocytes were seeded onto 12-well culture plates. They were serum-starved in Opti-MEM™ Medium for 12 h when they reached 80–90% confluence. Transfection was performed using the Lipofectamine 3,000 transfection reagent according to the manufacturer’s instructions provided in the manual. After 12 h of post-transfection incubation, the medium was replaced with adipogenic induction medium consisting of DMEM/F12 supplemented with 10% FBS (PAN-Biotech, USA), 1% penicillin/streptomycin, 0.5 mM IBMX (Sigma, St. Louis, MO, USA), 1 μM dexamethasone (DEX; Sigma, St. Louis, MO, USA), and 5 μg/mL bovine insulin (Sigma, St. Louis, MO, USA) to initiate differentiation. The following parameters were analyzed on days 4 and 8 of differentiation: mRNA and protein expression levels of *PLIN*1, *HSL*, and *ATGL*; lipid content absorbance; FFA content; and LD size. Differentiation rates were determined by microscopic cell counting using a net micrometer and dividing the number of differentiated adipocytes by the total number of cells.

### 2.4 Low temperature treatment of yak perirenal preadipocytes

After 9 days of differentiation induction, the differentiated adipocytes were subjected to either normal temperature (37 ± 1 °C) or low temperature (31 ± 1 °C) for 24 h and 72 h. The mRNA expression levels of *PLIN*1, *HSL*, *ATGL*, *PGC*-1*α*, cytochrome c oxidase subunit 7A1 (*COX*7*A*1), and *UCP*1 were analyzed, and the protein expression level of UCP1 was also detected.

### 2.5 Morphological observation of perirenal adipose tissue

Adipose tissue samples were fixed in 4% paraformaldehyde for 24 h, dehydrated through a graded ethanol series, embedded in paraffin, and sectioned at a thickness of 6 μm. Hematoxylin and eosin (H&E) staining was performed according to standard protocols. Briefly, sections were dewaxed, rehydrated, stained with H&E, dehydrated, and mounted for long-term preservation.

For immunofluorescence and immunohistochemistry, antigen retrieval was performed by heat treatment in a sodium citrate buffer (10 mM sodium citrate, 0.05% Tween 20, pH 6.0). Sections were blocked with 5% bovine serum albumin in Tris-buffered saline containing 0.1% Tween 20 (TBST, pH 7.6) for 30 min at 37 °C, followed by incubation overnight at 4 °C with the following primary antibodies: anti-PLIN1 (Bioss, bs-10779R, 1:500) and anti-UCP1 (Abcam, ab10893, 1:1000). After washing, sections were incubated with appropriate fluorescence- or horseradish peroxidase (HRP)-conjugated secondary antibodies for 30 min at 37 °C in the dark. Nuclei were counterstained with DAPI or hematoxylin, and the sections were dehydrated and covers lipped. Negative controls were processed identically except that the primary antibody was omitted. The stained sections were imaged using an inverted fluorescence microscope (Keyence BZ-X710) or a conventional light microscope (Olympus, Tokyo, Japan).

For Oil Red O staining, tissues were embedded in frozen medium (Leica) and sectioned at 6 μm, followed by staining with Oil Red O solution. LDs were visualized under a microscope (Olympus, Tokyo, Japan) and LD size was quantified using ImageJ software. For each age group, five perirenal adipose tissue sites were randomly selected from each calf. Six non-overlapping fields were randomly selected from each section and examined under a light microscope at 200× magnification. The morphology of the adipocytes was evaluated, and representative images were captured for analysis.

Transmission electron microscopy (TEM) of adipose tissue was performed as previously described (9).

### 2.6 RT-qPCR

Total RNA was extracted from the samples and adipocytes using TRIzol reagent (Invitrogen) according to the instructions of the manufacturer. Subsequently, 1 μg of RNA was reverse-transcribed to cDNA using the SuperScript™ III First-Strand Synthesis System (Invitrogen) according to the provided protocol. Quantitative real-time PCR (qPCR) was performed with SYBR® Green PCR Master Mix (Takara, Beijing, China) and on a 7,500 Real-Time PCR System (Applied Biosystems, Foster City, CA, USA). The expression of target genes was normalized to that of the endogenous control gene glyceraldehyde-3-phosphate dehydrogenase (GAPDH), and fold changes were calculated using the comparative CT method (2^−ΔΔCT^). The sequences of all the primers used are listed in Table 1. GAPDH was used as the internal reference gene for normalization. The stability of GAPDH expression across all experimental groups was evaluated and no significant variation was observed.

**Table 1:** The primer information of mRNA expression used in this study.

Primer names	Primer pairs (5′–3′)	Length (bp)	Annealing temp. (°C)
*PLIN1*	F: CTCCTGAAAAGATTGCCTCT	104	55
	R: GATTTTGTCTGAAGTGCTGG		
*HSL*	F: GGTAGAATTGGCTCAGGCGG	136	56
	R: TTGGACCCCTCTTGAACCCT		
*ATGL*	F: GTACCTGATGATACGCGCCA	133	58
	R: CAGCACCTCGCTGTAGGC		
*UCP1*	F: TGCGTGGCTGACATAATCACCTTC	115	55
	R: GGACACCTTTATACCTAATGGCACTGG		
*PGC1-a*	F: ATGGAGCAATAAAGCGAAGAGCATTTG	101	56
	R: GAGGAGGGTCATCATTTGTGGTCAG		
*COX7A1*	F: CAGAAACTCTTCCAGGAGGACA	98	59
	R: CCCAGACACAGAGTCATCGT		
*PPARa*	F: TGGAGATGGTGGACACAGAGAGC	94	58
	R: TCTTGTAGGAAGTCTGCCGAGAGC		
*GAPDH*	F: GCAAGTTCAACGGCACAGTCAAG	80	55
	R: TCGCTCCTGGAAGATGGTGATGG		

### 2.7 Western blotting

Total protein was extracted using ice-cold lysis buffer and a mechanical homogenizer (Tissue Tearor, Biospec Productions Inc., Bartlesville, OK, USA). Protein samples (20 μg per lane) were separated by 10% SDS-PAGE and subsequently transferred to a polyvinylidene difluoride (PVDF) membrane. The membranes were incubated overnight at 4 °C with the following primary antibodies: anti-PLIN1 (Bioss, bs-10779R, 1:500); anti-HSL (Proteintech, 17,333-1-AP, 1:4000); anti-ATGL (Zenbio, R23392, 1:500); and anti-UCP1(Abcam, ab10893, 1:1000). After incubation, the membranes were washed three times with phosphate-buffered saline containing Tween 20 (PBST) and incubated with the appropriate horseradish peroxidase (HRP)-conjugated secondary antibody for 2 h at room temperature. Protein bands were visualized using a supersensitive West Femto ECL chemiluminescence reagent (Thermo Scientific, USA) and imaged using a VersaDoc 2000 system (Bio-Rad, USA). Band intensities were quantified using ImageJ software (National Institutes of Health, Bethesda, MD, USA). Anti-β-actin antibody (AC026, Abclonal, 1:5000) was used as the loading control.

### 2.8 Cellular FFA content and absorbance determination

Preadipocytes were seeded into six-well cell culture plates. After 9 days of differentiation induction, the culture medium was collected, and FFA content was quantified using a microplate reader. The cells were washed three times with PBS, followed by fixation with 4% paraformaldehyde in PBS for 30 min. Fixed cells were stained with Oil Red O working solution for 30 min at room temperature, ensuring complete coverage of the bottom of each well. LDs were observed under a microscope (Olympus, Tokyo, Japan). Subsequently, isopropanol was added to each well for 10 min to elute the stain, and the absorbance at 510 nm was measured using a microplate reader.

### 2.9 Statistical analysis

Data were analyzed using SPSS software (Ver.17.0, SPSS Inc., Chicago, IL, USA). For the *in vivo* experiments, each yak calf was considered an independent biological replicate (*n* = 4 per group). For the *in vitro* experiments, assays were performed in four independent experiments with technical replicates. One-way ANOVA was used to test whether significant differences existed among the groups. Values in the figures are presented as mean ± SEM. The normality of the data distribution was evaluated using the Shapiro–Wilk test before performing ANOVA. *p* < 0.05 denoted statistical significance.

## 3 Results

### 3.1 Identification of yak perirenal adipocytes morphology

**Figure 2:**
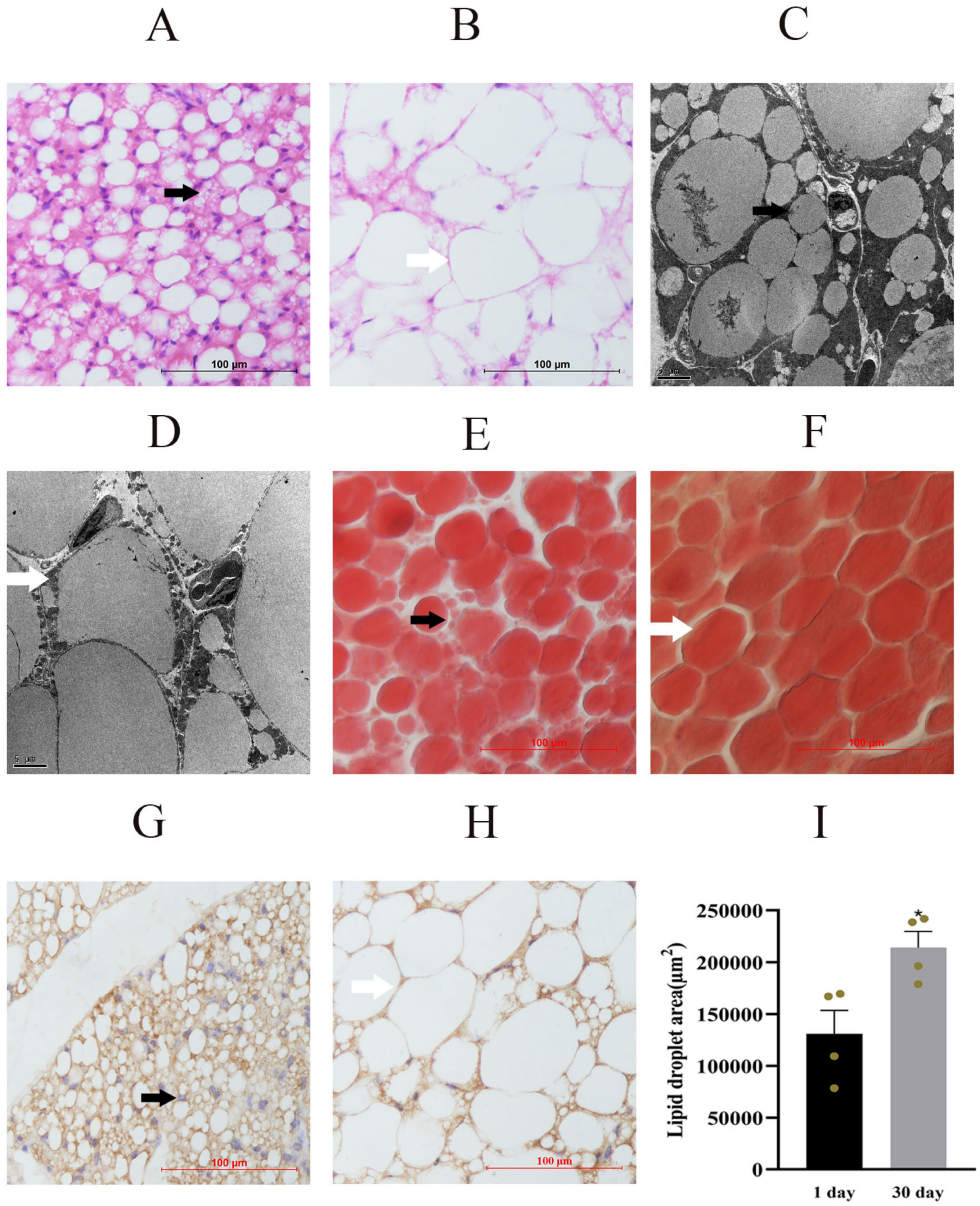
Morphological characteristics of adipocytes in perirenal adipose tissue from 1- and 30-days-old yak calves: **(A,B)** Hematoxylin and eosin (H. E.) staining of perirenal adipocytes at 1 **(A)** and 30 **(B)** days old. **(C,D)** SEM images visualizing LDs and mitochondria in perirenal adipocytes at 1 **(C)** and 30 **(D)** days old. **(E,F)** Oil Red O staining of LDs in perirenal adipocytes at 1 **(E)** and 30 **(F)** days old. **(G,H)** Immunohistochemical staining of UCP1 distribution in perirenal adipocytes at 1 day old **(G)** and 30 day old **(H)**. **(I)** LD areas were analyzed using ImageJ software. Black arrows indicate brown adipocytes and small LDs; white arrows indicate unilocular white adipocytes and large LDs. Scale bars: 100 μm **(A,B,E–H)**; 5 μm **(C,D)**. Data represent mean ± SEM from four independent experiments. Significance was defined as *p < 0.05, and ns indicates not significant.

H&E staining results indicated that multilocular adipocytes were predominant in the perirenal adipose tissue of yak calves at 1 day old (Figure 2A). However, some of them had transformed into unilocular adipocytes at 30 days of age (Figure 2B). Ultrastructural analysis and Oil Red O staining further demonstrated that the adipocytes in this tissue initially contained numerous small LDs on day 1 (Figures 2C,E). In contrast, the LDs were enlarged and vacuolated after 30 days, and they occupied almost the entire cytoplasmic space (Figures 2D,F). Immunohistochemical staining identified UCP1-positive adipocytes in both brown and white adipocyte populations within the perirenal adipose tissue (Figures 2G,H). Statistical analysis of the LD area confirmed that the LD in the perirenal adipocytes of 30-day-old yak calves was significantly larger than that in 1-day-old yak calves (Figure 2I). Together, these findings suggest that brown adipocytes containing small multilocular LDs differentiate into white adipocytes characterized by a single large unilocular LD in yak calves during the first month after birth.

### 3.2 Distribution and expression of PLIN1 in yak perirenal adipose tissues

**Figure 3:**
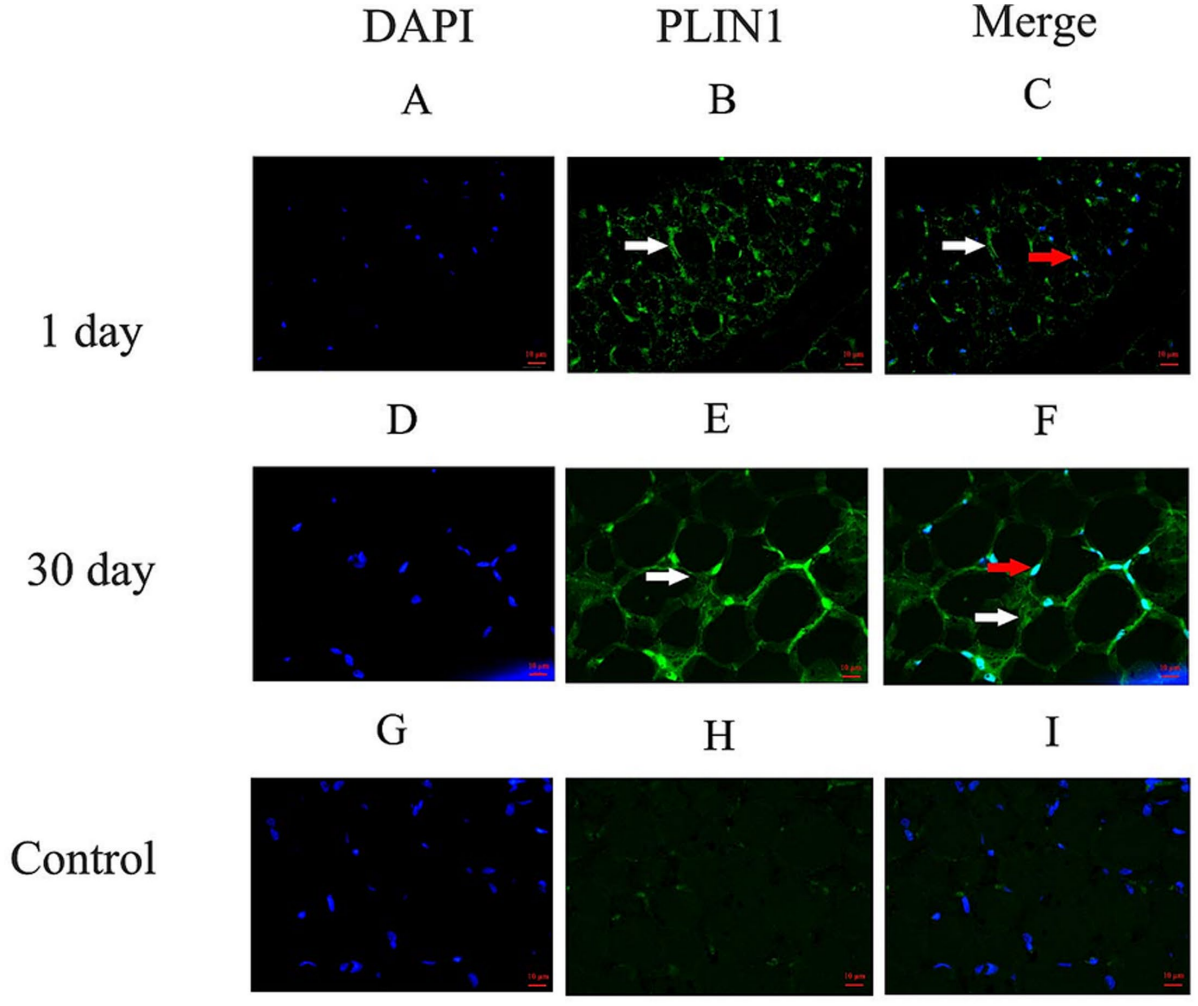
Immunofluorescence localization of PLIN1 on LDs in perirenal adipose tissue of 1- and 30-days-old yak calves: **(A–C)** Perirenal adipose tissue at 1 day old; **(D–F)** Perirenal adipose tissue at 30 days old; **(G–I)** Negative controls. White arrows indicate PLIN1-positive on LD in adipocytes, red arrows indicate nuclei of PLIN1-positive adipocytes. Scale bars: 10 μm.

To investigate the functional role of the PLIN1 gene and its encoded protein Perilipin 1 in yak perirenal adipose tissue, we assessed the distribution and expression levels of PLIN1 using immunofluorescence, RT-qPCR, and Western blotting. As shown in Figure 3, immunofluorescence analysis indicated that PLIN1 was specifically localized on the surface of lipid droplets (LDs) in both multilocular (Figures 3A–C) and unilocular (Figures 3D–F) adipocytes. In contrast, no obvious fluorescence signal was detected in the negative control group (Figures 3G–I), confirming the specificity of the PLIN1 antibody.

**Figure 4:**
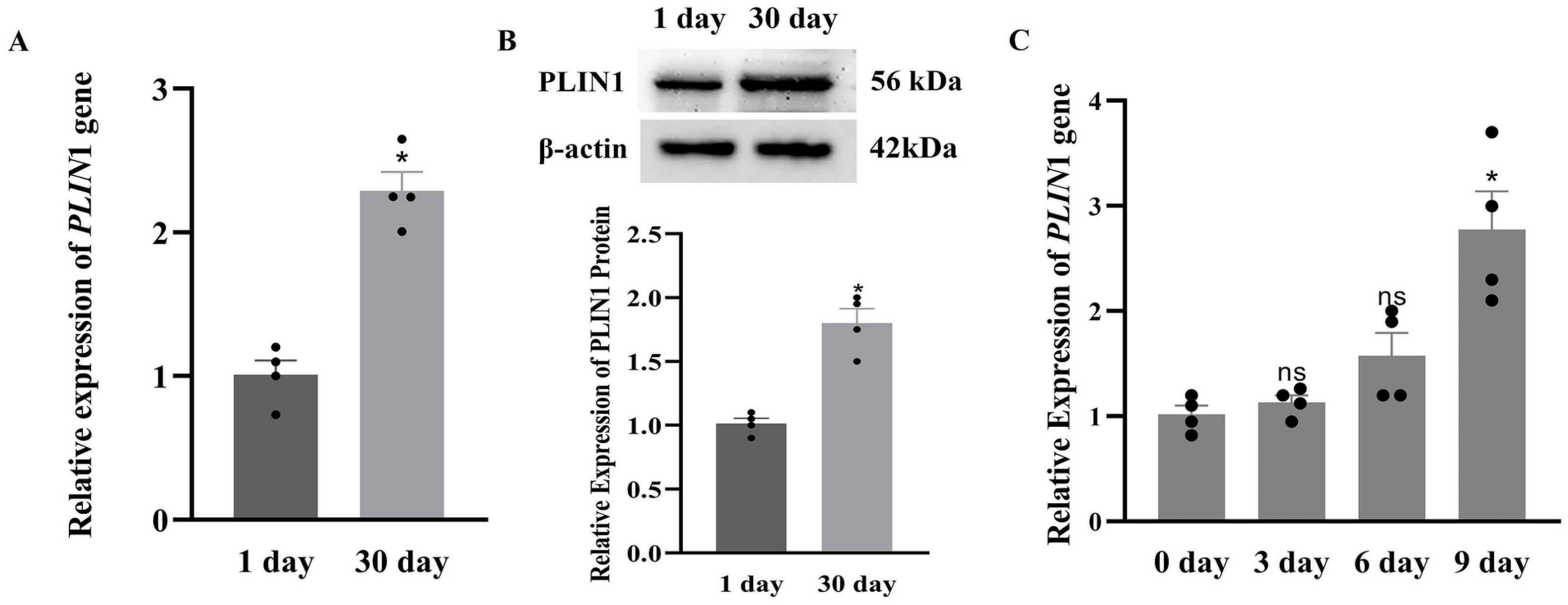
Expression of PLIN1 in perirenal adipose tissue of 1 and 30-days-old yak calves: **(A)** *PLIN*1 mRNA expression levels were determined by RT-qPCR; **(B)** PLIN1 protein expression levels were determined by western blotting; **(C)** Expression pattern of *PLIN*1 during differentiation of yak perirenal preadipocytes. Data represent mean ± SEM from four independent experiments. Significance was defined as *p < 0.05, and ns indicates not significant.

Quantitative PCR and Western blot analyses were performed to determine the expression levels of PLIN1 in adipocytes. As shown in Figure 4, both mRNA and protein expression levels of PLIN1 were significantly higher in 30-day-old than in 1-day-old yak calves (Figures 4A,B; *p* < 0.05).

### 3.3 Expression of PLIN1 in yak perirenal preadipocytes

To systematically investigate the temporal expression pattern of PLIN1 during yak perirenal preadipocyte differentiation, an *in vitro* differentiation model was established. Preadipocytes were cultured and induced to differentiate over 9 days. *PLIN*1 expression levels were assessed at 3-day intervals. Total RNA was extracted from cells on days 0, 3, 6, and 9 of differentiation, and *PLIN*1 mRNA expression was quantified. The results revealed time-dependent upregulation of *PLIN*1 expression throughout the differentiation process, with mRNA levels reaching a significant peak on day 9 (Figure 4C).

### 3.4 PLIN1 increases LD sizes, fat accumulation, and FA content in yak preadipocytes

**Figure 5:**
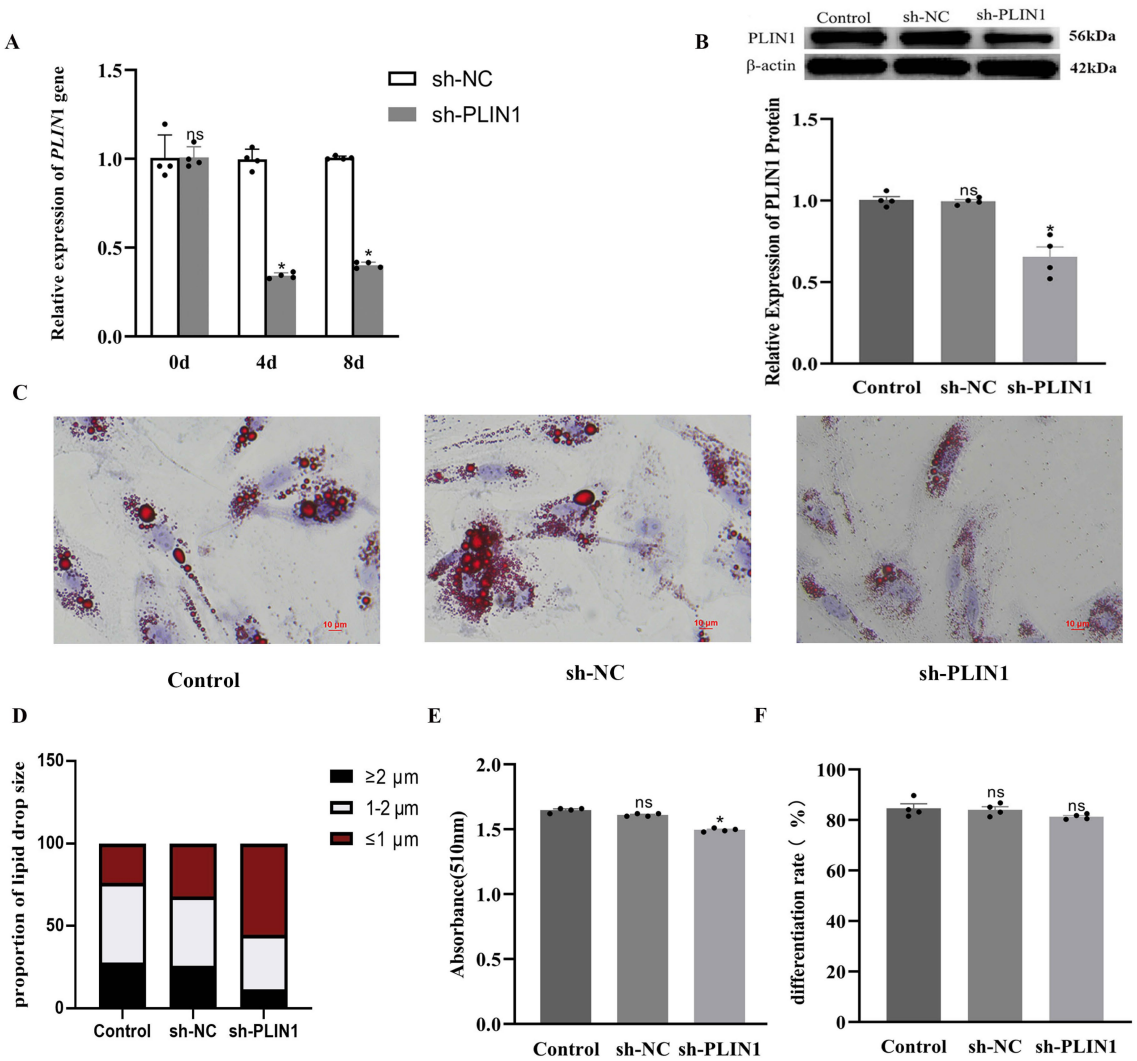
PLIN1 regulates lipid accumulation and LD formation in yak adipocytes: **(A)** PLIN1 mRNA expression after gene interference. **(B)** PLIN1 protein expression following gene interference. **(C)** LDs stained with Oil Red O. Scale bars: 10 μm. **(D)** Quantification of the percentage of lipid droplet size using Image J software. **(E)** Absorbance of lipid content measured at 510 nm. **(F)** The adipogenic differentiation rate. Data are presented as mean ± SEM (*n* = 4 independent experiments). *p < 0.05; ns, not significant.

To further elucidate the function of *PLIN*1 in yak adipocytes, we performed adenovirus-mediated knockdown using sh-*PLIN*1, with sh-NC as a negative control. Total RNA was collected on days 0, 4, and 8, and total protein was extracted on day 9 post-transfection. Knockdown of *PLIN*1 resulted in a significant reduction in both PLIN1 mRNA and protein levels (Figures 5A,B), confirming successful *PLIN*1 depletion.

Subsequent functional analyses showed that *PLIN*1 interference significantly reduced the percentage of lipid droplets with sizes of 1–2 μm and ≥2 μm, while increasing the proportion of lipid droplets ≤1 μm (Figures 5C,D). The lipid content also decreased relative to that in the control group (Figure 5E). No significant difference in the adipogenic differentiation rate was observed between the sh-PLIN1 and sh-NC groups, indicating that PLIN1 knockdown did not affect adipocyte differentiation (Figure 5F). These findings indicated that PLIN1 plays a critical role in promoting LD expansion and facilitating lipid accumulation in yak adipocytes.

### 3.5 PLIN1 affected lipid metabolism and fatty acid content in yak preadipocytes

**Figure 6:**
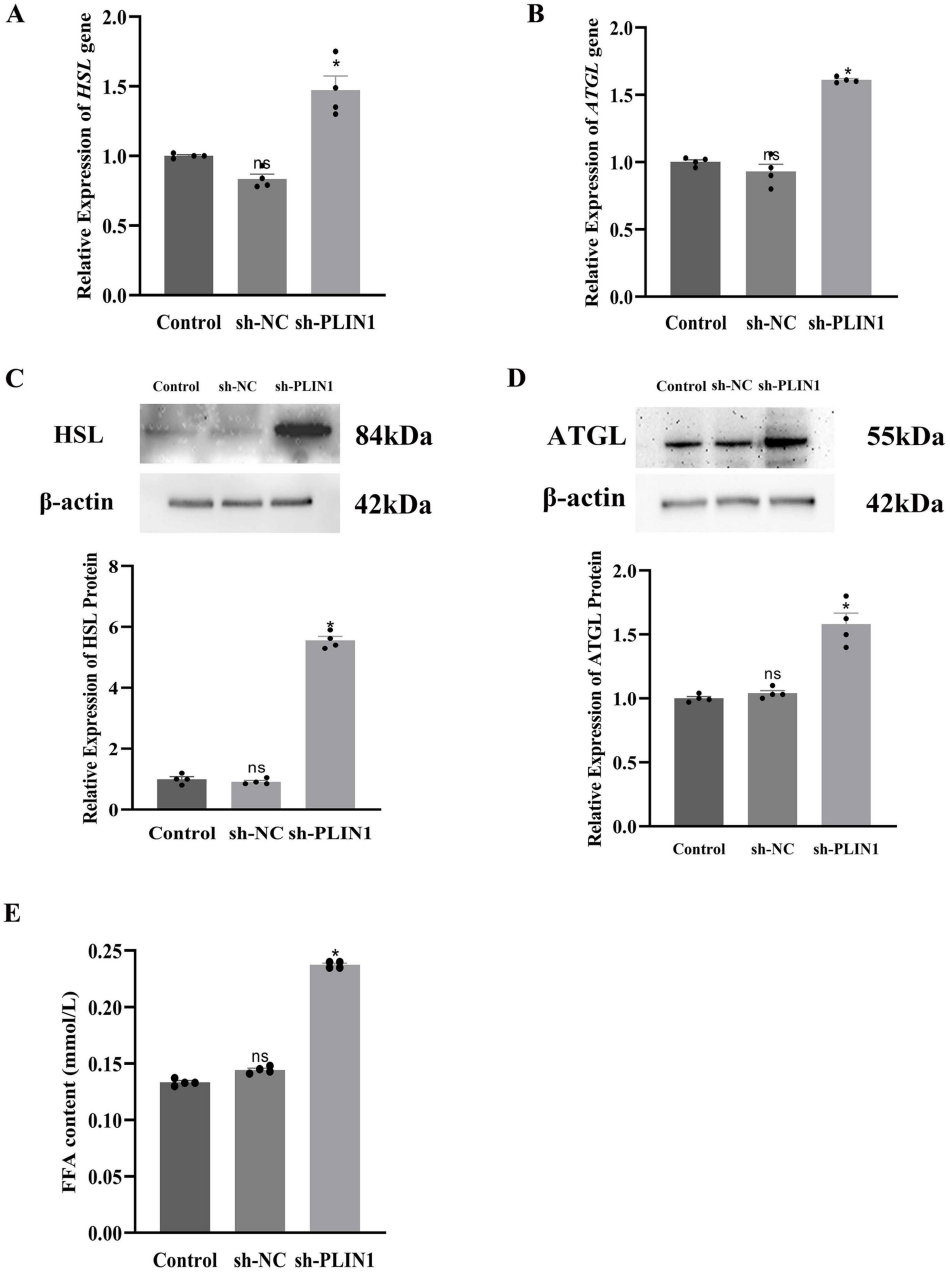
Effect of PLIN1 interference on FFA content and expression of HSL and ATGL expression in yak adipocytes: **(A)** mRNA expression levels of *HSL* following PLIN1 interference, determined by RT-qPCR; **(B)** mRNA expression levels of *ATGL* following PLIN1 interference, determined by RT-qPCR; **(C)** Protein expression levels of HSL following PLIN1 interference, determined by Western blotting; **(D)** Protein expression levels of ATGL following PLIN1 interference, determined by Western blotting; **(E)** FFA content after PLIN1 interference; Data represent the mean ± SEM of four independent experiments. *p < 0.05, and NS indicates no significant difference.

To further investigate the role of *PLIN*1 in lipid accumulation, the expression levels of key lipid metabolism-related genes were assessed on day 4 of yak perirenal preadipocytes differentiation. Following *PLIN*1 knockdown, the expression of ATGL and HSL were further enhanced at both mRNA and protein levels (Figures 6A–D). Consistent with this enhanced lipolytic profile, the content of FFA also increased after PLIN1 interference (Figure 6E). These findings indicate that PLIN1 modulates lipid accumulation by suppressing the expression of critical lipolytic enzymes such as ATGL and HSL and, in turn, reducing fatty acid release.

### 3.6 Cold exposure promotes PLIN1-mediated lipolysis and thermogenic activation in adipocytes

**Figure 7:**
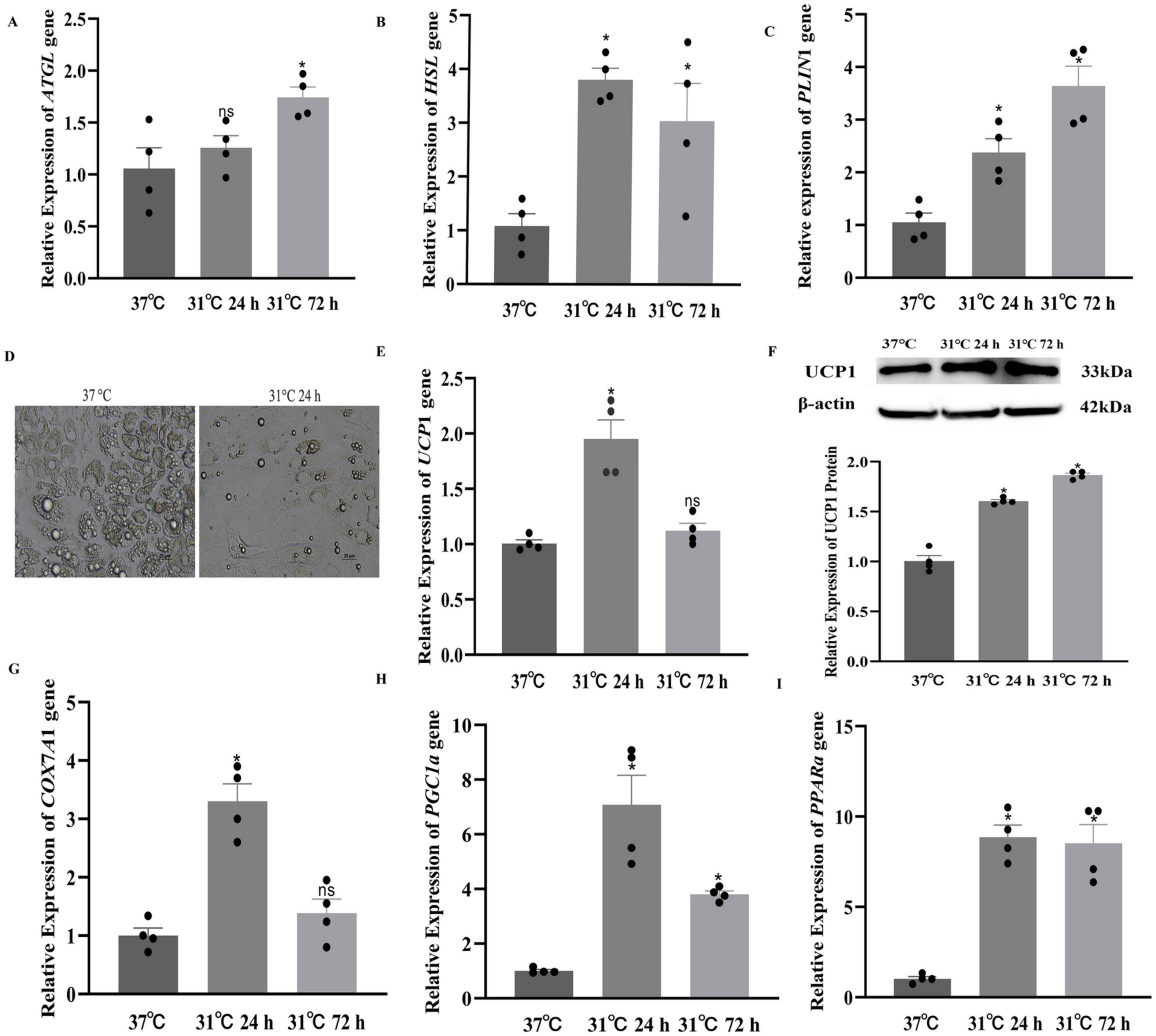
Effect of cold treatment on *HSL* and *ATGL* mRNA expression in yak adipocytes: **(A)** *ATGL* gene expression; **(B)** *HSL* gene expression; **(C)** *PLIN*1 gene expression; **(D)** Adipocyte morphology. Scale bars: 20 μm; **(E)** *UCP*1 gene expression; **(F)** Protein abundance of UCP1 in response to cold exposure was assessed via western blot analysis; **(G)** *COX*7*A*1 gene expression; **(H)** *PGC*-1*α* gene expression; **(I)** *PPARα* gene expression. Data are mean ± SEM of four independent experiments. *p < 0.05, and ns indicates no significant difference.

To elucidate the molecular mechanism through which PLIN1 regulates thermogenesis and activation in brown adipocytes, we evaluated the mRNA expression levels of ATGL and HSL under cold exposure. Adipocytes subjected to 31 °C for 24 and 72 h showed significantly higher mRNA levels of *ATGL*, *HSL*, and *PLIN*1 than the control cells (Figures 7A–C). Moreover, the adipocytes had morphological features consistent with white adipose tissue browning after 72 h of cold stimulation (Figure 7D), and the expressions of the thermogenesis-related genes (*UCP*1, *COX*7*A*1, *PGC*-1*α*, and *PPARα*) significantly increased (Figures 7E,G–I). In addition, western blot analysis confirmed that the protein abundance of UCP1 significantly increased following cold exposure (Figure 7F). These findings indicated that PLIN1 enhanced lipolysis to support thermogenic activation in beige adipocytes.

## 4 Discussion

The presence of several small multilocular LDs, high mitochondrial density, and elevated expression of UCP1 are established hallmark features of brown adipocytes (6, 7). In this study, we observed a substantial population of small adipocytes in the perirenal adipose tissue of 1-day-old yak calves. They had abundant small LDs, numerous mitochondria, and high UCP1 expression. These morphological and molecular features indicate the presence of classical brown adipocytes in neonatal yaks, which are likely specialized for thermogenesis and energy expenditure during early postnatal development, a pattern consistent with observations in other species such as calves (10–12), goats (13, 14), mice (27, 28), and humans (29).

In contrast, the perirenal adipose tissue predominantly contained large white adipocytes with a single unilocular LD, reduced mitochondrial content, and low UCP1 expression at 30 days of age. These observations suggest that a phenotypic transition occurs during the first month of life, when some brown adipocytes differentiate into white adipocytes. This shift aligns with the reported postnatal developmental changes in other ruminants such as goats (13, 14) and calves (10–12), as well as in the interscapular and subcutaneous adipose tissues of yak calves (9). However, the mechanisms underlying the transdifferentiation of brown adipocytes into white adipocytes in postpartum yak calves remain unclear. We propose that this process is closely associated with the progressive growth and fusion of small multilocular LDs into larger unilocular droplets during adipocyte maturation. However, the molecular mechanisms regulating LD dynamics and unilocular LD formation in adipocytes remain poorly understood. Further investigations are necessary to elucidate the signaling pathways and key regulatory factors involved, which may provide important insights into lipid metabolism and adipose tissue plasticity in large mammals. Recent studies have emphasized that lipid droplet dynamics and adipose tissue remodeling are closely associated with systemic metabolic regulation and disease processes, highlighting the broad biomedical relevance of lipid metabolism research (30).

Previous studies have indicated that the LD-associated protein PLIN1 promotes LD fusion and formation of large LDs (31). In the present study, we observed that LDs in the perirenal adipose tissue of 30-day-old yak calves were larger than those in 1-day-old calves, accompanied by a significantly higher expression of PLIN1 at both the mRNA and protein levels. Furthermore, PLIN1 expression was upregulated during adipocyte differentiation, suggesting a positive correlation between PLIN1 expression and LD size, which is consistent with the findings in bovine (20) and chicken adipocytes (21). To investigate the regulatory role of PLIN1 in LD dynamics, we performed PLIN1 knockdown *in vitro*. Interference with PLIN1 expression resulted in reduced LD size and lipid accumulation, and the adipogenic differentiation rate, as evaluated by the proportion of LD-positive cells, was not significantly altered following PLIN1 knockdown. This suggests that PLIN1 does not affect adipocyte differentiation per se but primarily regulates lipid droplet growth and lipid storage during adipocyte maturation, indicating that PLIN1 promotes the fusion of small LDs and formation of large LDs during adipocyte differentiation. This is consistent with reports that PLIN1 increases triglyceride synthesis and promotes the formation of large LDs (20). Moreover, PLIN1 interference led to upregulated transcript levels and protein abundance of the lipolytic enzymes HSL and ATGL, along with an increase in FFA content. Taken together with the *in vivo* observations of increased LD size and PLIN1 expression between postnatal days 1 and 30, these results suggested that PLIN1 suppresses basal lipolysis in white adipocytes by inhibiting HSL and ATGL. During periods of energy deprivation, hydrolysis of TG stored in WAT is stimulated to release FFA into the circulation, enabling other organs to utilize them as an energy source (24). This finding further supports the hypothesis that PLIN1 inhibits basal lipolysis and contributes to energy storage in 30-day-old yak calves.

Interestingly, our study found that the expressions of PLIN1, HSL, and ATGL were significantly upregulated during 24–72 h of cold exposure. This is consistent with reports that cold exposure increases PLIN1 expression, which participates in classical lipid metabolism pathways to provide energy for cold adaptation in the inguinal fat of Hezuo pigs (18). This also aligns with observations that mouse BAT started expressing the LD structural protein PLIN-2/ADRP, showed increased expression of PLIN1 and elevated levels of both HSL and ATGL in LDs (19).

Additionally, our results showed that cold exposure induced morphological changes consistent with white fat browning (beige adipocyte formation) and significantly increased the expression of thermogenesis-related genes (*UCP*1, *PPARα*, *PGC*-1*α*, and *COX*7*A*1). Consistently, UCP1 protein abundance was markedly elevated, further confirming the activation of thermogenic programs in adipocytes under cold stimulation. Therefore, our findings indicate that PLIN1 may facilitate lipolysis in adipocytes by activating HSL and ATGL, which supports thermogenesis in response to cold exposure. Thus, the high expression of PLIN1 in beige adipocytes may serve a dual function: maintaining LD stability under basal conditions and promoting lipolysis and thermogenesis under stimulatory conditions such as cold exposure.

In summary, we found that PLIN1 is associated with lipid droplets and mediates lipolysis through HSL and ATGL in the perirenal brown adipose tissue of yak calves. However, the mechanism by which PLIN1 interacts with HSL to regulate lipolysis in the adipose tissues of yak calves remains unclear. Previous studies have shown that Apole6 disrupts the PLIN1-HSL interaction to inhibit lipolysis (24), while TP53INP2 actively promotes lipophagy by targeting LDs through interaction with PLIN1 in murine 3T3-L1 cells (32). Further investigation is required to clarify the regulatory mechanisms underlying PLIN1-mediated lipolysis in yaks.

## 5 Conclusion

In this study, we observed that perirenal adipocytes in 1-day-old yak calves contained multilocular small lipid droplets (LDs), which had fused into large unilocular LDs by 30 days of age. PLIN1 was expressed in both brown and white adipocytes, and it had significantly higher mRNA and protein levels in 30-day-old than in 1-day-old calves. During *in vitro* perirenal adipocyte differentiation, *PLIN*1 mRNA expression peaked on day nine. Inhibition of *PLIN*1 resulted in reduced LD size and lipid accumulation, along with increased FFA content and upregulation of both mRNA and protein expression of ATGL and HSL. Additionally, cold exposure elevated the expressions of *PLIN*1, *HSL*, *ATGL*, *UCP*1, *PPAR*α, *PGC*-1*α*, and *COX*7*A*1. These findings offer new insights for future investigations into the molecular mechanisms underlying brown-to-white phenotypic transition during early postnatal development in yaks.

## Data Availability

The datasets presented in this study can be found in online repositories. The names of the repository/repositories and accession number can be found in the article/supplementary material.
